# Tampa Scale for Kinesiophobia in chronic neck pain patients (TSK-neck): structural and construct validity and reliability in a Brazilian population

**DOI:** 10.1186/s12891-024-07268-6

**Published:** 2024-02-17

**Authors:** Letícia Padilha Mendes, Cid André Fidelis-de-Paula-Gomes, André Pontes-Silva, Felipe Souza Barreto, Jocassia Silva Pinheiro, Aron Charles Barbosa da Silva, Flávio de Oliveira Pires, Plinio da Cunha Leal, Mariana Arias Avila, Almir Vieira Dibai-Filho

**Affiliations:** 1 Department of Physical Education, Universidade Federal do Maranhão, São Luís, Brazil; 2 Postgraduate Program in Rehabilitation Sciences, Universidade Nove de Julho, São Paulo, Brazil; 3 Postgraduate Program in Physical Therapy, Universidade Federal de São Carlos, São Carlos, Brazil; 4 Postgraduate Program in Physical Education, Universidade Federal do Maranhão, São Luís, Brazil; 5 Sarah Network of Rehabilitation Hospitals, São Luís, Brazil; 6 Postgraduate in Rehabilitation and Functional Performance, Faculdade de Medicina de Ribeirão Preto, Universidade São Paulo, Ribeirão Preto, Brazil

**Keywords:** Fear of movement, Neck pain, Factor analysis, Surveys and questionnaires

## Abstract

**Background:**

To date, there are no studies in the literature that define the internal structure of the Tampa Scale for Kinesiophobia (TSK) in patients with chronic neck pain based on factorial analysis. As such, we aimed to verify and identify the best structure of the Brazilian version of the TSK in patients with chronic neck pain.

**Methods:**

We included Brazilian participants aged ≥18 years, both sexes, with self-reported neck pain for more than 3 months and pain intensity ≥3 on the Numerical Pain Rating Scale (NPRS). Dimensionality and number of TSK items were assessed using confirmatory factor analysis (CFA). We tested the following internal structures: structure 1 (1 domain and 17 items), structure 2 (1 domain and 11 items), structure 3 (2 domains and 11 items), and structure 4 (2 domains and 9 items). We used the Pain-Related Catastrophizing Thoughts Scale (PCTS) and the NPRS for construct validity. In addition, we assessed test-retest reliability for the seven-day interval using intraclass correlation coefficient (ICC_2,1_), Cronbach’s alpha to assess internal consistency, and ceiling and floor effects.

**Results:**

The study sample included of 335 patients. Most were women (77.6%), young adults (~ 34 years), single (48.4%), with complete primary education (57.3%), physically inactive (66.6%), with a mean pain duration of 46 months and a mean pain intensity of ~ 5 points on the NPRS. Redundancy was found in the following items: item 1 with item 2 (modification indices = 21.419) and item 13 with item 15 (modification indices = 13.641). Subsequently, based on these paired analyses, the items with the lowest factor loadings (items 2 and 15) were excluded. As such, TSK structure 4 was composed of two domains (“somatic focus” and “activity avoidance”) and 9 items, which showed adequate fit indices and lower AIC and SABIC values. We observed significant values (*p* < 0.05) with a correlation magnitude greater than 0.142 to 0.657 between the two domains of the TSK-neck and the other instruments (PCTS and NPRS). We found excellent reliability (ICC_2,1_ ≥ 0.96) and adequate internal consistency (Cronbach’s alpha ≥0.98) of the TSK-neck. Finally, ceiling and floor effects were not observed.

**Conclusion:**

The TSK-neck structure with two domains (somatic focus and activity avoidance) and nine items is the most appropriate for patients with chronic neck pain.

**Supplementary Information:**

The online version contains supplementary material available at 10.1186/s12891-024-07268-6.

## Background

Neck pain, a common musculoskeletal disorder, is associated with several physical, psychosocial, and individual risk factors [1]. Regarding psychosocial factors, an important tool to assess fear of movement is the Tampa Scale for Kinesiophobia (TSK). It is a self-report measure consisting of 17 items and 1 domain, developed in English in 1990. Scores range from 17 to 68 points. The higher the score, the greater the degree of kinesiophobia, indicating that the individual is afraid of movement [2].

The TSK has already been translated, adapted, and validated for several languages, such as Dutch [3], French [4], Norwegian [5], Spanish [6], and Swedish [7]. In Brazil, the validation study was developed by Siqueira et al. [8] in low back pain patients and showed good potential for clinical application, but did not meet the suggestions of the Rasch model. To the best of our knowledge, the use of the TSK in neck pain showed acceptable reliability and construct validity in the Persian [9] and Japanese versions [10].

A study [11] evaluated the measurement properties of the TSK in people with neck pain and, using Rasch analysis, identified a structure with 1 domain and 11 items as adequate, concluding that an instrument is a good option for assessing fear of movement. Recently, a study in Brazil performed confirmatory factor analysis and found a valid internal structure of the TSK with 9 items and 2 domains (activity avoidance and somatic focus). However, it was applied only to patients with chronic low back pain [12].

To date, there are no studies in the literature that define the internal structure of the TSK in patients with chronic neck pain based on factorial analysis. Therefore, due to the importance of the correct use of this instrument, the present study aimed to verify and identify the best structure of the Brazilian version of the TSK in patients with chronic neck pain.

## Methods

### Study design and ethical aspects

A cross-sectional study of the structural validity of the TSK. Data collection took place in physiotherapy clinics in the city of São Luís (Maranhão, northeast of Brazil). In addition, an online platform was used to collect data from patients with neck pain throughout the country (Brazil). This study was previously approved by the research ethics committee of each institution (report number 3.182.525).

### Sample size and eligibility criteria

The sample size followed the recommendations according to the Consensus-based Standards for the Selection of Health Measurement Instruments (COSMIN) [13]. Namely, seven times the number of items in the questionnaire. Therefore, we set a minimum of 119 participants to conduct the present study based on the 17-item TSK. In addition, we recruited a sub-sample to check the test and retest reliability of the TSK-neck, with a sample size based on the COSMIN recommendations (*n* = 50) [14].

We included individuals of both sexes, age ≥ 18 years, self-reported neck pain ≥3 points on the Numerical Pain Rating Scale (NPRS), and pain duration ≥3 months. We excluded individuals with a history of tumors, cervical fractures, infectious diseases, physiotherapeutic treatment of the cervical region in the past 3 months, the presence of neurological disorders involving the central nervous system, and psychiatric changes that would make it impossible to complete the questionnaire.

### Assessments

We performed an initial assessment that included personal, sociodemographic, anthropometric and clinical aspects. We used the Brazilian version of the NPRS [15] to characterize participants’ pain intensity on a one-dimensional scale from 0 to 10 points, where 0 represents “no pain” and 10 represents “worst pain imaginable”.

The TSK is a self-administered scale consisting of 17 questions about pain and symptom intensity. The score varies from one to four points, with a score of 1 indicating “strongly disagree”, 2 indicating “somewhat disagree”, 3 indicating “somewhat agree”, and 4 indicating “strongly agree”. To obtain the final total score, it is necessary to invert the scores of questions 4, 8, 12, and 16. The final score can be a minimum of 17 points and a maximum of 68 points. Higher scores indicate a greater degree of kinesiophobia [8]. In the present study, we used the version of the TSK translated and adapted in Brazil by Siqueira et al. [8].

### Statistical analysis

Regarding the descriptive statistical analysis (quantitative and categorical), we present the values as mean, standard deviation, absolute number, and percentage. We then performed confirmatory factor analysis (CFA) to identify the best TSK structure using R Studio software (Boston, MA, USA), using the packages lavaan and semPlot, as well as the implementation of a polychoric matrix and the robust diagonally weighted least squares (RDWLS) extraction method [16, 17]. We considered adequate values in the fit indices for the following cutoff points: chi-square/degrees of freedom (DF) < 3, comparative fit index (CFI) and Tucker-Lewis index (TLI) > 0.90, and root mean square error of approximation (RMSEA) < 0.08 [17, 18].

When comparing the different models, lower values of the Akaike information criterion (AIC) and the sample-size adjusted Bayesian information criterion (SABIC) indicated the most appropriate structure [19]. Factor loadings were considered adequate with values greater than 0.40 [20]. We tested 4 TSK structures: 1) the original 17-item unidimensional structure proposed for the Brazilian version of the TKS [21]; 2) the reduced 11-item unidimensional structures [11, 22]; 3) the 11-item two-dimensional structure [23]; and 4) the 9-item two-dimensional structure generated here using Modification Indices (MI), a questionnaire refinement resource within CFA.

We used the Pain-Related Catastrophizing Thoughts Scale (PCTS) [24] and the NPRS [15] for construct validity, with the hypothesis that there would be a positive Spearman correlation (from 0.30 to 0.50) between the two domains of the TSK-neck (activity avoidance and somatic focus) and the other instruments (PCTS [24] and NPRS [15]). In addition, we assessed test-retest reliability for the seven-day interval using intraclass correlation coefficient (ICC_2,1_), standard error of measurement (SEM), and minimal detectable change (MDC) [25]. For interpretation of ICC_2,1_ values: less than 0.40 = poor; between 0.40 and 0.75 = moderate; between 0.75 and 0.90 = substantial; and greater than 0.90 = excellent [26]. Cronbach’s alpha was also used to assess internal consistency, with values between 0.70 and 0.95 indicating good internal consistency [27].

Finally, we also assessed ceiling and floor effects. By definition, these effects occur when a number of study participants (more than 15%) reach the minimum or maximum value of the questionnaire, indicating a problem in assessing the responsiveness of the instrument [28].

## Results

The study sample included of 335 patients. Most were women (77.6%), young adults (~ 34 years), single (48.4%), with complete primary education (57.3%), physically inactive (66.6%), with a mean pain duration of 46 months and a mean pain intensity of ~ 5 points on the NPRS (Table 1).

**Table 1:** Clinical characteristics of participants with neck pain (*n* = 335)

Variable	Number (%) or mean (standard deviation)
Sex (female)	260 (77.6%)
Age (years)	34.22 (12.61)
Body mass (kg)	67.68 (12.49)
Stature (m)	1.64 (0.09)
Body mass index (kg/m^2^)	25.04 (3.84)
Marital status	
Single	162 (48.4%)
Married	156 (46.6%)
Divorced	11 (3.3%)
Widower	6 (1.8%)
Level of education	
Complete primary education	192 (57.3%)
Complete secondary education	43 (12.8%)
Complete higher education	100 (20.9%)
Physical Activity (no)	223 (66.6%)
Smoker (no)	325 (97%)
Pain chronicity (months)	46.93 (40.26)
Numerical Pain Rating Scale (score, 0–10)	5.42 (2.17)
Tampa Scale for Kinesiophobia (TSK-neck)	
Activity avoidance domain (score, 4–16)	10.25 (2.84)
Somatic focus domain (score, 5–20)	11.96 (3.44)

Table 2 shows the structures tested. First, we tested the 1-domain, 17-item version of the TSK (structure 1) and the 1-domain, 11-item version of the TSK (structure 2), but all fit indices showed inadequate values (chi-square/DF > 3, TLI and CFI < 0.90, and RMSEA > 0.08). Subsequently, we obtained the version with two domains (“somatic focus” and “activity avoidance”) and 11 items as Structure 3, and all fit indices were also inadequate.

**Table 2:** Comparison among the internal structures of the Tampa Scale for Kinesiophobia (*n* = 335)

Structures	Chi-square/DF	CFI	TLI	RMSEA (90% CI)	AIC	BIC
Structure 1	6.28	0.687	0.642	0.126 (0.117, 0.135)	15,762.366	15,892.046
Structure 2	4.98	0.846	0.807	0.109 (0.095, 0.124)	10,260.713	10,274.837
Structure 3	4.25	0.877	0.843	0.099 (0.084, 0.114)	10,232.692	10,320.417
Structure 4	2.75	0.950	0.931	0.072 (0.053, 0.093)	8351.278	8423.747

Structure 1: 1 domain and all 17 items; Structure 2: 1 domain and 11 items (1, 2, 3, 5, 6, 7, 10, 11, 13, 15 and 17); Structure 3: 2 domains and 11 items (domain 1: items 1, 2, 10, 13, 15 and 17; domain 2: items 3, 5, 6, 7 and 11); Structure 4: 2 domains and 9 items (domain 1: items 1, 10, 13 and 17; domain 2: items 3, 5, 6, 7 and 11)

*DF* degree of freedom: *CFI* comparative fit index: *TLI* Tucker-Lewis index: *RMSEA* root mean square error of approximation: *CI* confidence interval: *AIC* Akaike information criterion: *BIC* Bayesian information criterion

**Fig. 1:**
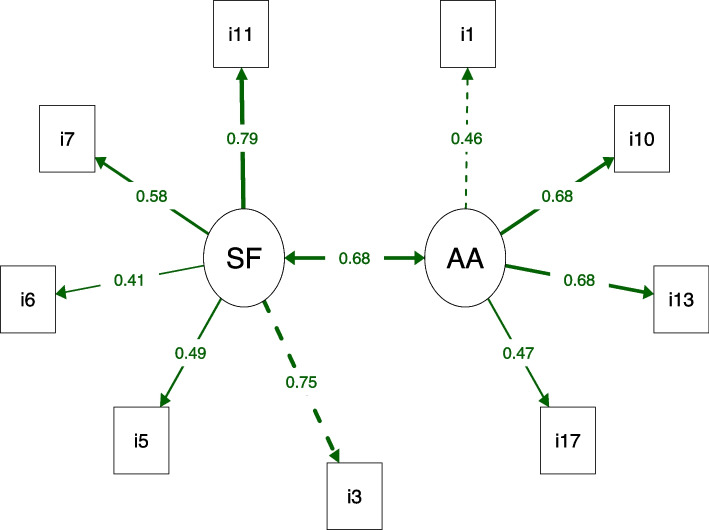
Path diagram of the nine-item Tampa Scale for Kinesiophobia with values representing factor loadings between domains and their items. AA: Activity avoidance domain; SF: Somatic focus domain. The dotted line indicates the first item in the domain

Starting from structure 3, we use the MI to identify the problems of this structure. Redundancy was found in the following items: item 1 with item 2 (MI = 21.419) and item 13 with item 15 (MI = 13.641). Subsequently, based on these paired analyses, the items with the lowest factor loadings (items 2 and 15) were excluded. As such, TSK structure 4 was composed of two domains (“somatic focus” and “activity avoidance”) and 9 items, which showed adequate fit indices and lower AIC and SABIC values (Table 2). Furthermore, as shown in Fig. 1, the factor loadings between domains and items were adequate (> 0.40). This correct TSK structure (called TSK-neck) is available in Additional file.

Regarding the construct validity, we observed significant values (*p* < 0.05) with a correlation magnitude greater than 0.142 to 0.657 between the two domains of the TSK-neck (activity avoidance and somatic focus) and the other instruments (PCTS and NPRS) (Table 3). Table 4 shows the characteristics of the neck pain sub-sample used for test and retest reliability.

**Table 3:** Correlation between Tampa Scale for Kinesiophobia (TSK-neck) domains and other instruments for construct validity (*n* = 335)

Variables	Activity avoidance domain	Somatic focus domain
Numerical Pain Rating Scale		
At rest	rho = 0.142, *p* = 0.031 *	rho = 0.318, *p* < 0.001 *
Ater movement	rho = 0.173, *p* = 0.009 *	rho = 0.288, *p* < 0.001 *
Pain-Related Catastrophizing Thoughts Scale	rho = 0.399, *p* < 0.001 *	rho = 0.657, p < 0.001 *

rho: Spearman’s correlation; * Significant correlation (*p* < 0.05)

**Table 4:** Neck pain sub-sample used for test-retest reliability (*n* = 50)

Variable	Number (%) or mean (standard deviation)
Sex (female)	41 (82%)
Age (years)	33.74 (10.79)
Body mass (kg)	69.79 (16.07)
Stature (m)	1.64 (0.07)
Body mass index (kg/m^2^)	25.95 (5.20)
Physical Activity (no)	20 (40%)
Smoker (no)	49 (98%)
Pain chronicity (months)	63.94 (59.91)
Numerical Pain Rating Scale at test (score, 0–10)	4.52 (1.99)
Numerical Pain Rating Scale at retest (score, 0–10)	4.36 (1.68)

Table 5 shows the excellent reliability (ICC_2,1_ ≥ 0.96) and adequate internal consistency (Cronbach’s alpha ≥0.98) of the TSK-neck. For the activity avoidance domain, we observed that 2.1 and 2.4% of participants achieved the minimum (4) and maximum (16) scores, respectively. For the somatic focus domain, 3.9 and 1.5% of participants achieved the minimum (5) and maximum (20) scores, respectively. Thus, ceiling and floor effects (< 15%) were not observed.

**Table 5:** Reliability of the Tampa Scale for Kinesiophobia (TSK-neck) sub-sample (*n* = 50)

Domain	Test	Retest	ICC_2,1_	95% CI	SEM	MDC	Cronbach’s α
AV	10.36 (2.70)	10.36 (2.77)	0.965	0.939, 0.980	0.51	1.42	0.982
SF	11.08 (3.42)	11.04 (3.49)	0.974	0.954, 0.985	0.65	1.79	0.987

Test and retest shown as mean (standard deviation)

*ICC*_2,1_ intraclass correlation coefficient: *CI* confidence interval: *SEM* standard error of measurement: *MDC* minimum detectable change: *AV* Activity avoidance domain: *SF* Somatic focus domain

## Discussion

Our study found that the most appropriate internal structure for the TSK-neck has two domains (“somatic focus” and “activity avoidance”) and 9 items. The other structures tested (17-item unidimensional, 11-item unidimensional, and 11-item bidimensional) showed inadequate fit indices in the factor analysis.

In general, proposed patient-reported outcome measures should be evaluated and adapted for different populations and specific diseases [29]. Due to this scientific path, several versions of the same instrument are published each year until the best version is found for a specific sample. For example, the structure of the TSK has been shown to be unstable in several studies: in patients with low back pain, Rosenbloom et al. [30] state that the 13-item TSK is reliable and probably the most appropriate; Woby et al. [22] describe an 11-item unidimensional TSK; Tkachuk et al. [31], Al-Shudifat et al. [32], and Roelofs et al. [23] identified an 11-item, two-domain TSK; and finally, Pontes-Silva et al. [12] suggest that a two-domain (activity avoidance and somatic focus), nine-item structure is most appropriate.

In contrast, only one study has evaluated the internal structure of the TSK in patients with neck pain of primarily traumatic origin, identifying the unidimensional internal structure with 11 items as adequate by Rasch analysis, but proposing a new calculation of the scale score based on a transformation matrix [11]. In our study of patients with chronic neck pain, we tested this internal structure (structure 2) previously proposed in the literature [11], but all the fit indices were inadequate, so this internal structure was rejected. Due to this new proposal, as well as the lack of studies similar to ours, new studies must focus their efforts on confirming a valid internal structure for the TSK neck that remains stable regardless of the culture and language of the population.

In terms of clinical applicability, it is essential that the constructs assessed by a scale are clear and well defined. In addition, the items of a scale must not be redundant or discrepant. Thus, factor analysis via structural equation modeling identifies the best relationships between latent variables and their items [16]. Therefore, the 9-item two-dimensional version has clear constructs and is short enough to be completed quickly, in addition to having enough items to adequately assess the domains. In addition, the TSK-neck structure has substantial reliability and internal consistency, with established standard errors of measurement and minimal detectable change, which strengthens the recommendation for its use in the clinical context [26].

Finally, regarding the limitations of the study, we conducted the analysis on a sample of Brazilian participants. In fact, it is crucial that new studies with samples from other countries test the nine-item TSK-neck with two domains. In addition, other measurement properties such as reliability, construct validity, and responsiveness should also be considered.

## Conclusion

The TSK-neck structure with two domains (somatic focus and activity avoidance) and nine items is the most appropriate for patients with chronic neck pain.

### Supplementary Information

**Supplementary Material 1.**

## Data Availability

The data and materials in this paper are available from the corresponding author on request.

## Abbreviations

AIC: Akaike Information Criterion
BIC: Bayesian Information Criterion
CI: Confidence interval
CFA: Confirmatory Factor Analysis
CFI: comparative fit index
COSMIN: COnsensus based Standards for the selection of health Measurement INstruments)
DF: degree of freedom
LEFS: Lower Extremity Functional Scale
NPRS: 11-item Numeric Pain Rating Scale
RDWLS: Robust Diagonally Weighted Least Squares
rho: Spearman’s Correlation Coefficient
RMSEA: Root Mean Square Error of Approximation
SRMR: Standardized Root Mean Squared Residual
TLI: Tucker Lewis index
TSK: Tampa Scale for Kinesiophobia
