# Supplementary material for: Tampa Scale for Kinesiophobia in chronic neck pain patients (TSK-neck): structural and construct validity and reliability in a Brazilian population
Source: BMC Musculoskelet Disord. 2024 Feb 17;25:151. doi: 10.1186/s12891-024-07268-6 (PMC10874046; doi:10.1186/s12891-024-07268-6)
Supplement: Supplementary file 1 — Supplementary Material 1. [file 12891_2024_7268_MOESM1_ESM.pdf]

English version of the Tampa Scale for Kinesiophobia for chronic neck pain patients (TSK-neck)

Here are some things other patients told us about their pain. For each statement, provide a score from 1 to 4 in case you agree or disagree with the statement.

**Items**	**Strongly disagree**	**Somewhat disagree**	**Somewhat agree**	**Strongly agree**
Activity avoidance domain				
1. I'm afraid that I might injury myself if I exercise.	1	2	3	4
10. Simply being careful that I do not make any unnecessary movements is the safest thing I can do to prevent my pain from worsening.	1	2	3	4
13. Pain lets me know when to stop exercising so that I don't injure.	1	2	3	4
17. No one should have to exercise when he/she is in pain.	1	2	3	4
Somatic focus domain				
3. My body is telling me I have something dangerously wrong.	1	2	3	4
5. People aren't taking my medical condition seriously enough.	1	2	3	4
6. My accident has put my body at risk for the rest of my life.	1	2	3	4
7. Pain always means I have injured my body.	1	2	3	4
11. I wouldn't have this much pain if there weren't something potentially dangerous going on in my body.	1	2	3	4

Versão brasileira da Tampa Scale for Kinesiophobia para cervicalgia crônica (TSK-neck)

Aqui estão algumas das coisas que outros(as) pacientes nos contaram sobre a dor deles(as). Para cada afirmativa, por favor, indique um número de 1 a 4, caso você concorde ou discorde da afirmativa.

Itens	Discordo totalmente	Discordo parcialmente	Concordo parcialmente	Concordo totalmente
Domínio evitação de atividades				
1. Eu tenho medo de me machucar se eu fizer exercícios.	1	2	3	4
10. A atitude mais segura que posso tomar para prevenir a piora da minha dor é, simplesmente, ser cuidadoso para não fazer nenhum movimento desnecessário.	1	2	3	4
13. A dor me avisa quando devo parar o exercício para eu não me machucar.	1	2	3	4
17. Ninguém deveria fazer exercícios quando está com dor.	1	2	3	4
Domínio foco somático				
3. Meu corpo está me dizendo que alguma coisa muito errada está acontecendo comigo.	1	2	3	4
5. As pessoas não estão levando minha condição médica a sério.	1	2	3	4
6. Minha lesão colocou o meu corpo em risco para o resto da minha vida.	1	2	3	4
7. A dor sempre significa que o meu corpo está machucado.	1	2	3	4
11. Eu não teria tanta dor se algo realmente perigoso não estivesse acontecendo no meu corpo.	1	2	3	4
